# Effects of donor/recipient human leukocyte antigen mismatch on human cytomegalovirus replication following liver transplantation

**DOI:** 10.1111/tid.12325

**Published:** 2015-01-09

**Authors:** RW Aldridge, FM Mattes, N Rolando, K Rolles, C Smith, G Shirling, C Atkinson, AK Burroughs, RSB Milne, VC Emery, PD Griffiths

**Affiliations:** 1 Department of Infection and Population Health, University College London (UCL)London, UK; 2 UCL HospitalsLondon, UK; 3 The Royal Free Sheila Sherlock Liver Centre, Royal Free HospitalLondon, UK; 4 Research Department of Infection and Population Health, UCLLondon, UK; 5 Anthony Nolan Centre, Anthony Nolan TrustLondon, UK; 6 UCL/Medical Research Council Centre for Medical Molecular VirologyLondon, UK; 7 Department of Microbial and Cellular Sciences, University of SurreySurrey, UK

**Keywords:** cytomegalovirus, liver transplant, HLA match, immunity

## Abstract

**Background:**

Natural immunity against cytomegalovirus (CMV) can control virus replication after solid organ transplantation; however, it is not known which components of the adaptive immune system mediate this protection. We investigated whether this protection requires human leukocyte antigen (HLA) matching between donor and recipient by exploiting the fact that, unlike transplantation of other solid organs, liver transplantation does not require HLA matching, but some donor and recipient pairs may nevertheless be matched by chance.

**Methods:**

To further investigate this immune control, we determined whether chance HLA matching between donor (D) and recipient (R) in liver transplants affected a range of viral replication parameters.

**Results:**

In total, 274 liver transplant recipients were stratified according to matches at the HLA A, HLA B, and HLA DR loci. The incidence of CMV viremia, kinetics of replication, and peak viral load were similar between the HLA matched and mismatched patients in the D+/R+ and D−/R+ transplant groups. D+/R− transplants with 1 or 2 mismatches at the HLA DR locus had a higher incidence of CMV viremia >3000 genomes/mL blood compared to patients matched at this locus (78% vs. 17%; *P* = 0.01). Evidence was seen that matching at the HLA A locus had a small effect on peak viral loads in D+/R− patients, with median peak loads of 3540 and 14,706 genomes/mL in the 0 and combined (1 and 2) mismatch groups, respectively (*P* = 0.03).

**Conclusion:**

Overall, our data indicate that, in the setting of liver transplantation, prevention of CMV infection and control of CMV replication by adaptive immunity is minimally influenced by HLA matching of the donor and recipient. Our data raise questions about immune control of CMV in the liver and also about the cells in which the virus is amplified to give rise to CMV viremia.

Cytomegalovirus (CMV) remains an important opportunistic pathogen in transplant patients. After liver transplantation, the virus is associated with a range of direct and indirect effects including hepatitis and acute rejection (1, 2). Currently, CMV disease can be prevented during the immediate post-transplant period by giving antiviral drugs, either prophylactically from the time of transplant, or preemptively upon detection of CMV viremia (3).

Post transplant, CMV-seropositive recipients (R+) can reactivate latent CMV as a consequence of immunosuppression. In addition, the virus can be transmitted with a donor liver to cause primary infection in a CMV-seronegative individual (R−) or reinfection in a CMV-seropositive individual (4). The donor/recipient (D/R) CMV serostatus combinations are associated with different risks of CMV viremia post transplant, D+/R− giving the highest risk, followed by D+/R+, then D−/R+ and D−/R−. Although it is well established that CMV disease is less frequent in those with prior natural immunity, it is not clear whether this protective immunity is conferred by cytotoxic T lymphocytes or other components of the adaptive or innate immune systems. However, CMV disease predominantly occurs in patients with suppressed T-cell immunity and recent data have shown that the quality of the CD4 and CD8 response is correlated with the control of high-level replication and consequent prevention of CMV disease after liver, kidney, and heart-lung transplantation (5–8).

Studies of the role of CD8 T cells in controlling CMV after liver transplantation are confounded by the fact that transplant donors and recipients are matched on blood-group but not on human leukocyte antigen (HLA) type, although HLA matching can occur by chance. CD8+ T cell-mediated control of CMV infection in a transplanted liver is likely to be impaired when donor cells present CMV-derived peptides on class I HLA molecules, which are not matched to the recipient HLA type.

On this basis, we hypothesized that donors and recipients who were, by chance, matched at HLA loci would be better able to control CMV replication than those patients whose transplants were not HLA matched. In this study, we tested this hypothesis in a cohort of patients managed by preemptive therapy and found that control of CMV replication post transplant is minimally influenced by HLA matching between donor and recipient.

## Materials and methods

### Patients

All liver transplants (*n* = 331) performed from January 2003 to October 2008 at the Royal Free Hospital, London, UK were identified from the transplant database. Patients were excluded from the study if:

1. The CMV serostatus of donor or recipient before transplantation could not be confirmed (*n* = 5);
2. No CMV viral load data were available post transplant (*n* = 18);
3. They had received an experimental CMV vaccine during the study period (*n* = 16) as part of a clinical trial (9).

Patients with multiple transplants were included once only in their highest risk D/R CMV serostatus group (D+/R− > D+/R+ > D−/R+ > D−/R−; *n* = 18). This left a total of 274 patients in this historical cohort. All patients accepted into the liver transplant program gave written informed consent for their laboratory results to be analyzed for research purposes.

### Detection and management of CMV viremia

Whole blood samples for CMV polymerase chain reaction (PCR) were requested twice weekly from inpatients and at each subsequent outpatient visit (typically scheduled weekly for 4 weeks, then every 2 weeks thereafter).

Total nucleic acids were extracted from whole blood using the QIAamp DNA Blood Mini Kit (QIAGEN Ltd, Manchester, UK) until October 2006, and the Biomerieux EasyMag system thereafter (Biomérieux, Marcy L'Etoile, France). Real-time PCR for quantification of CMV DNA was performed on the TaqMan® 7700/7500 system (Applied Biosystems/Lifetechnologies, Grand Island, New York, USA) using an in-house assay targeting a 150-base pair amplicon from the CMV UL55 gene that encodes glycoprotein B (10). Serial dilutions of cloned amplicon (from 200 plasmid copies/mL to 2 × 10^6^ plasmid copies/mL) were run in triplicate on each plate as a standard for quantification. CMV load was calculated using Applied Biosystems sequence detection software. The detection limit was 200 genomes/mL (equivalent to 168 international units [IU]/mL) (11).

The standard clinical practice at the Royal Free Hospital for preemptive therapy was followed for CMV management throughout the study period and no patient received antiviral prophylaxis. In accordance with our routine protocol, if viral load exceeded 3000 genomes/mL in whole blood (equivalent to 2520 IU/mL), the patient was treated with twice-daily intravenous ganciclovir 5 mg/kg (or twice-daily oral valganciclovir 900 mg), with dose adjustment for renal function, until CMV DNA was undetectable (<200 genomes/mL) in 2 consecutive blood samples. We and others have previously shown that the kinetics of response of CMV load to ganciclovir and valganciclovir therapy are equivalent (12, 13).

### Virological outcome measures

Viral load measurements were obtained from the hospital pathology database for a 90-day period commencing on the day of transplantation. The following virological parameters were assessed:

1. Cumulative incidence of viremia >3000 genomes/mL in the 90 days post transplant.
2. Time to viremia post transplantation. Follow up began at the day of transplantation and person-time at risk was calculated for each patient as the number of days from transplantation until: (i) the last negative CMV PCR of the follow up; or (ii) the first CMV PCR >3000 genomes/mL; or (iii) death.
3. Trajectory of viremia (mean increase per day as a log_10_ function of viral load) in patients who experienced CMV viremia >3000 genomes/mL. First, a graph was plotted of log_10_ CMV load against time. The viral load time points defined in order to calculate the trajectory were: (i) last negative sample (<200 genomes/mL) prior to the start of the viremia; (ii) first positive (>200 genomes/mL); (iii) peak viral load. Viral loads in between first positive and peak viral load were also used to calculate the trajectory. A linear regression line was then fitted to these data, and the trajectory during the viremic episode was calculated using the regression coefficients. Only the first viremic episode for each patient was considered. Individuals without a negative sample at least 7 days before the first positive sample were excluded, as it would not be possible to accurately predict the start of viremia in these cases. Trajectory was reported as the mean increase in the viral load (log_10_ genomes/mL/day).
4. Peak viral load in all patients with detectable viremia (i.e., viral load >200 genomes/mL).

### HLA matching

For each outcome of interest, the effect of HLA matching at A, B, and DR alleles was examined. HLA typing was performed at the Anthony Nolan Centre using the microcytotoxicity technique as described previously (13). Mismatches were classified as either 0 mismatches, or 1 and 2 mismatches (combined into one group) for each of the HLA alleles.

### CMV serology

The results for each outcome were additionally stratified by the CMV immunoglobulin-G (IgG) status of donors and recipients by the following D/R serostatus groups: D−/R−, D−/R+, D+/R+, D+/R−. Recipient CMV IgG status before transplantation was determined using a Biomérieux VIDAS from the start of the cohort until July 2008, after which it was carried out on an Abbott Architect i2000 SR (Abbott Diagnostics, Abbott Park, Illinois, USA). For recipients with multiple samples before transplantation, IgG status was based on the sample taken closest to the date of transplantation. CMV serological testing of donors was performed by the host hospital and confirmed in house when samples were available.

### Data sources

Clinical and HLA data were extracted from a Microsoft Access database maintained by the liver transplant unit and CMV PCR data from the hospital pathology database. These 2 sets of data were then matched using the unique hospital number in Microsoft Access.

### Statistical analysis

Sample size of the cohort was determined by the transplantation numbers. Statistical analysis was performed using Stata version 11 (StataCorp LP, College Station, Texas, USA). Categorical variables were analyzed using chi-square or Fisher's exact test. Kruskal–Wallis tests were used to analyze numerical variables. Kaplan–Meier survival analysis was used for time-dependent analysis, with groups compared using the log-rank test. Assessment was made for missing data using simple descriptive statistics.

## Results

### Study participants

Of the 274 subjects included in the study, 102 were female and 172 male (Table 1). CMV IgG positivity was higher among transplant recipients (74%, 203/274) than donors (49%, 134/274). D/R HLA mismatches at 1 or 2 loci were most frequent for HLA B (90%, 246/274), followed by HLA A (85%, 233/274) and finally HLA DR (84%, 231/274). The total person-time at risk was 55.8 years, and the mean length of follow up was 74.4 days. The median number of samples per patient in the 90-days follow up post transplantation was 15 (interquartile range [IQR] 9, 24). No data were missing with respect to clinical or demographic details of the patients in the final dataset used for this analysis.

**Table 1:** Baseline characteristics of the study population

	Number	%
Total no. patients	274	100
Gender		
Male	172	63
Female	102	37
Median age at transplant (IQR)	51 (42–58)	
Recipient CMV positive	203	74
Donor CMV positive	134	49
D− R−	38	14
D+ R−	33	12
D− R+	102	37
D+ R+	101	37
Viremias within 60 days of follow up		
>3000 copies	54	20
>200 copies	110	40
HLA A mismatches		
0	41	15
1 & 2	233	85
HLA B mismatches		
0	28	10
1 & 2	246	90
HLA DR mismatches		
0	43	16
1 & 2	231	84

IQR, interquartile range; CMV, cytomegalovirus; D, donor; R, recipient; HLA, human leukocyte antigen.

### Effect of HLA matching on CMV replication

#### A. Occurrence of high-level CMV replication (>3000 genomes/mL)

Fifty-four patients (20%) had a viral load >3000 genomes/mL within 90 days of transplantation and therefore received antiviral therapy. A high level of CMV viremia during the first 90 days post transplant was most common in the D+/R− group (67%, 22/33), followed by the D+/R+ group (23%, 23/101), and finally the D−/R+ group (9%, 9/102 (*P* < 0.001, chi-square test for trend). No episodes of CMV viremia were detected in the 90-day period following transplantation for the D−/R− group.

**Fig 1:**
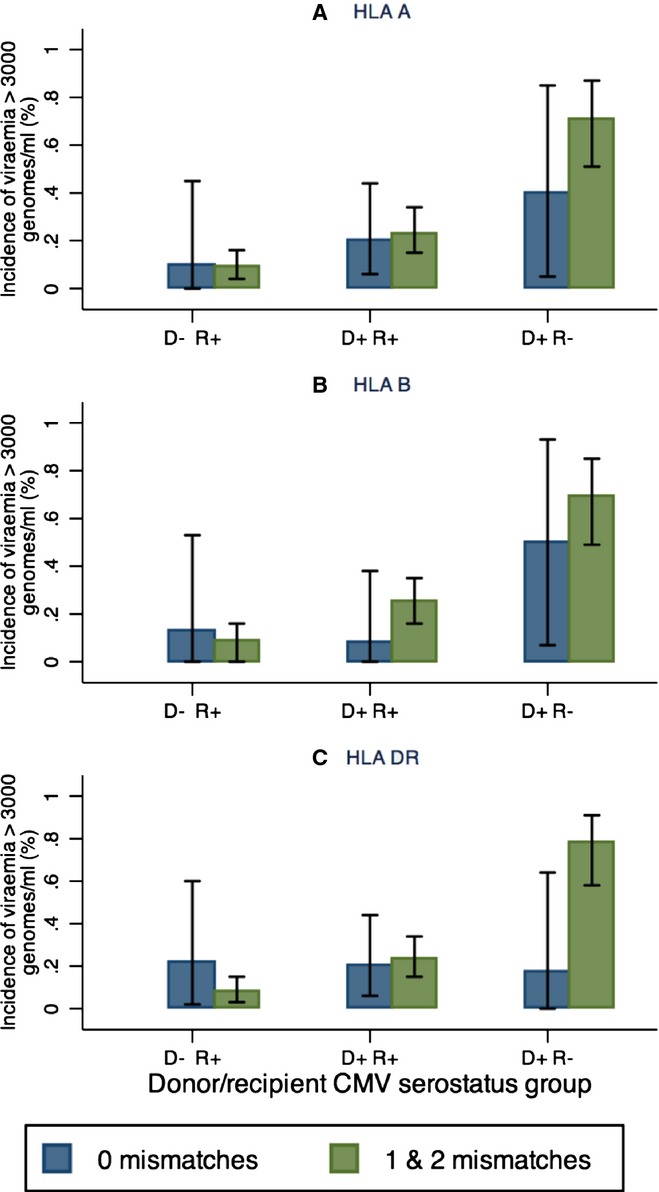
Impact of (A) human leukocyte antigen (HLA) A; (B) HLA B; and (C) HLA DR mismatch on incidence of high-level cytomegalovirus (CMV) viremia (>3000 genomes/mL) according to donor (D)/recipient (R) CMV serostatus. Proportion of patients in each group with high-level viremia is shown. Blue bars: no mismatch; green bars: 1 or 2 mismatches. Error bars show 95% confidence intervals.

D+/R− transplants with 1 or 2 mismatches at the HLA DR locus had a higher incidence of viremia >3000 genomes/mL (78%; 21/27), compared to patients matched at this locus (17% [1/6]; *P* = 0.01 Fisher's exact test; Fig.1, Table S1). No such difference was seen in the D+/R+ or D−/R+ groups. HLA matching at HLA A or B loci had no significant effect on the incidence of CMV viremia >3000 genomes/mL in any of the D/R groups.

#### B. Time to CMV viremia >3000 genomes/mL

**Fig 2:**
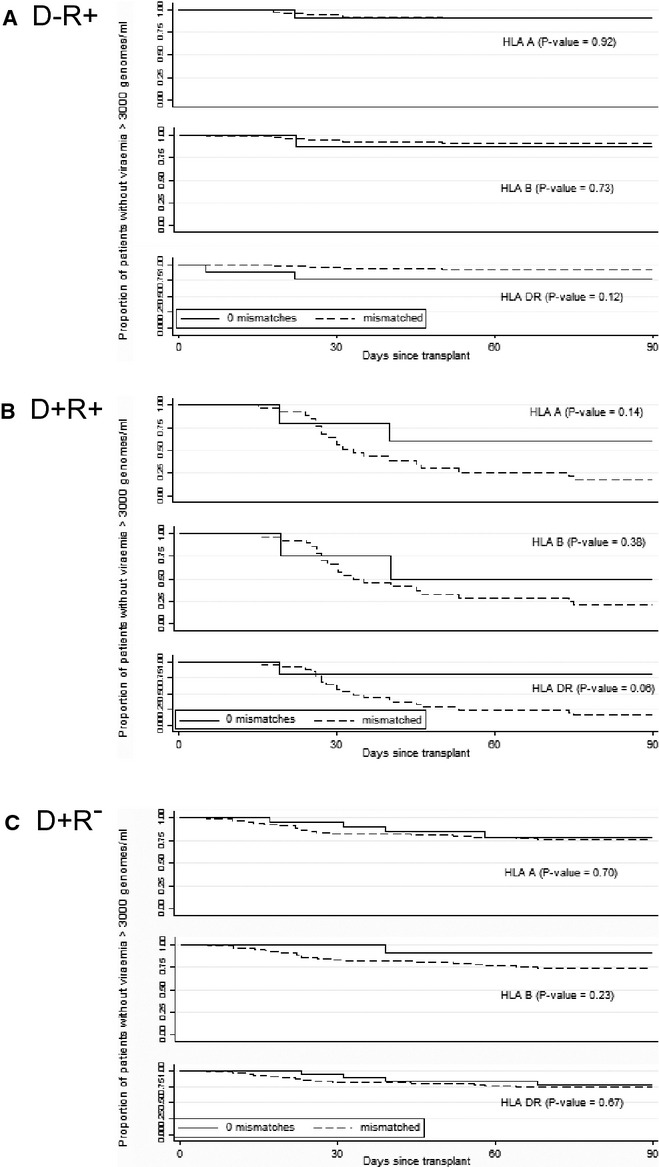
Kaplan–Meier analysis of the impact of human leukocyte antigen (HLA) A, B, and DR mismatch on the time elapsed between transplantation and onset of high-level viremia (>3000 genomes/mL) according to donor (D)/recipient (R) cytomegalovirus serostatus: (A) D−R+; (B) D+R+; (C) D+ R−.

Survival analysis was performed on the time to viremia post transplantation (Fig.2). In D−/R+ transplants, at risk of CMV reactivation, the proportion with viremia at 90 days was 0.12, (95% confidence interval [CI]: 0.07, 0.64) and 0.08 (95% CI: 0.04, 0.16), respectively in the 0 and combined HLA DR mismatched groups (*P* = 0.12, log-rank test, Fig.2A). The pattern was similar with HLA A or B mismatches (*P* = 0.92, and 0.73, respectively, log-rank test). In the D+/R+ transplants, at risk of reinfection or reactivation (Fig.2B), and the D+/R− transplants, at risk of primary infection (Fig.2C), matching at HLA A, B, or DR loci also had no effect on time to viremia (*P* = 0.14, and 0.38 and 0.06, respectively, for D+/R+; *P* = 0.70, 0.23 and 0.67, respectively, for D+/R−, log-rank test).

#### C. Trajectory of viral load

The trajectory of viral load was analyzed among the subgroup of individuals who experienced CMV viremia >3000 genomes/mL. The median trajectories for the D+/R−, D+/R+, and D−/R+ stratified groups were 0.17, 0.18, and 0.22 log_10_ genomes/mL per day, respectively (*P* = 0.36, Kruskal–Wallis test).

No evidence was seen for a difference in the trajectory of viral load with increases in the number of mismatches at the HLA A or DR loci (Table 2). A trend was noted suggesting that trajectories varied by HLA B mismatches in the D+/R− group, with median trajectories of 0.37 (IQR 0.34–0.40), 0.09 (IQR 0.05–0.20) in the 0 and combined 1 and 2 mismatch groups (*P* = 0.04, Kruskal–Wallis test; Table 2).

**Table 2:** Trajectory (log_10_ genomes/mL/day) of viremia in individuals with viremias >3000 genomes/mL according to donor (D)/recipient (R) cytomegalovirus status and human leukocyte antigen (HLA) mismatch level

	HLA mismatches	Total in group	Trajectory median log copies/mL/day (IQR)	*P*-value*
HLA A				
D− R+	0	0		–
	1 & 2	6	0.18 (0.15–0.32)	
D+ R+	0	1	0.34 (0.34–0.34)	0.16
	1 & 2	15	0.11 (0.07–0.18)	
D+ R−	0	1	0.34 (0.34–0.34)	0.22
	1 & 2	9	0.12 (0.06–0.23)	
HLA B				
D− R+	0	0		–
	1 & 2	6	0.18 (0.15–0.32)	
D+ R+	0	0		–
	1 & 2	16	0.12 (0.09–0.24)	
D+ R−	0	2	0.37 (0.34–0.40)	0.04
	1 & 2	8	0.09 (0.05–0.20)	
HLA DR				
D− R+	0	1	0.15 (0.15–0.15)	0.38
	1 & 2	5	0.2 (0.17–0.32)	
D+ R+	0	2	0.23 (0.15–0.31)	0.27
	1 & 2	14	0.11 (0.07–0.18)	
D+ R−	0	1	0.34 (0.34–0.34)	0.22
	1 & 2	9	0.12 (0.06–0.23)	

Numbers in this table do match those in Table 1, as it was not possible to calculate trajectory in each case owing to missing data.

* Kruskall–Wallis test.

IQR, interquartile range.

#### D. Peak viral load

As expected, peak viral load varied significantly according to D/R CMV status (*P* < 0.001, Kruskal–Wallis test). Median peak viral load was 12,332 genomes/mL in the D+/R− group, 2616 genomes/mL in the D+/R+ group, and 1047 genomes/mL in the D−/R+ group.

**Fig 3:**
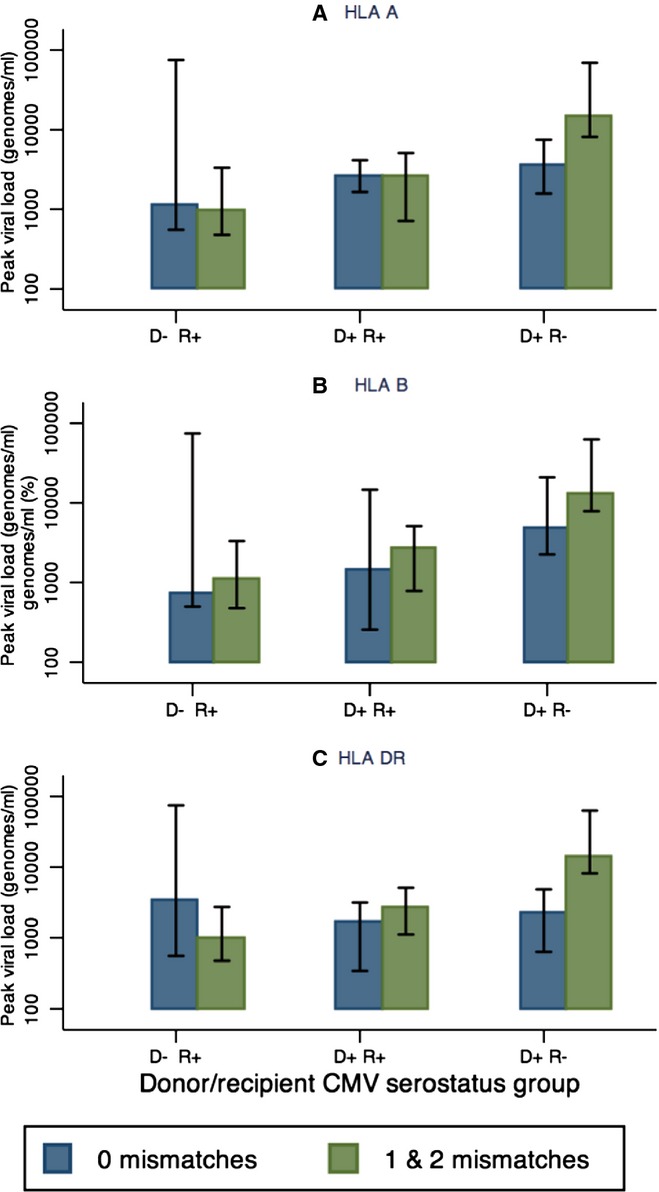
Impact of (A) human leukocyte antigen (HLA) A; (B) HLA B; and (C) HLA DR mismatch on peak viral load among all viremic (>200 genomes/mL) patients. Median peak viral load is plotted. Blue bars: no mismatch; green bars: 1 or 2 mismatches. Error bars show interquartile range. CMV, cytomegalovirus; D, donor; R, recipient.

Evidence indicates that matching at the HLA A locus had a small effect on peak viral loads in D+/R− patients (median peak loads: 3540 genomes/mL [IQR 1575–7496] and 14,706 genomes/mL [IQR 8131–68,854] in the 0 and combined [1 and 2] mismatch groups, respectively, *P* = 0.03). A trend was also seen for a similar effect when these D+/R− transplant recipients were stratified according to HLA DR and HLA B mismatches (Fig.3; Table S2). No effect was seen for mismatches at any HLA loci for the D−/R+ and D+/R+ groups.

## Discussion

The results presented herein confirm that pre-existing natural immunity can control CMV replication after transplantation (14), as illustrated by significantly fewer seropositive than seronegative recipients having viremia when challenged with seropositive donor organs, and by a significantly lower peak viral load. This control may reflect a range of immune effectors including antibodies, T cells, and NK cells. The present study indicates that HLA matching in liver transplantation explained only a small proportion of the protective effect conferred by pre-transplant immunity against CMV. The weak associations we observed were in the primary infection D+/R− group, where we saw an increased incidence of CMV viremia >3000 genomes/mL in the presence of mismatches at the HLA DR locus, and an elevated peak viral load in D+/R− patients when mismatches at the HLA A and HLA DR loci were present. A trend suggests that trajectories varied by HLA B mismatches in the D+/R− group. Our study combines data from a large transplant database at a single UK transplant center and, based upon the completeness of the dataset, minimal loss to follow up, and minimal chance of selection bias, the results support the general conclusion that HLA mismatch at the 3 loci examined does not have a major impact on the incidence or kinetics of CMV viremia after liver transplantation in patients with pre-existing immunity to CMV.

Microcytotoxicity was used to determine the HLA matching for this study. Although more advanced techniques may exist that would allow a greater level of differentiation for mismatches, this methodology is used in many clinical settings. We have therefore taken a pragmatic approach to use these data. It is important to note that we have not attempted to quantify the immunosuppressive regimens used in all the transplant patients included in the study; however, the transplant center from which the data are taken uses an established standard protocol in the majority of its patients.

It is notable that all of the effects we observed were in the D+/R− patient group. The elevated incidence of viremia in HLA DR mismatched patients in this group is consistent with an important role for CD4 T cells in control of primary CMV infection in this setting (7, 8, 15, 16). Direct CD4-mediated cytotoxicity is one possible mechanism for control, and we have shown previously that a decreased level of poly-functional CD4 T cells correlates with viremia after liver transplantation (7). Other studies have shown that, in this context, CD4 and CD8 T cells have high levels of PD-1 and are poorly proliferative (17–19). The role of CD4 T cells in generation of an antibody response to the primary infection is also of potential importance. Indeed, we recently showed that a vaccine-induced antibody response correlated with control of CMV viremia, and may have reduced transmission rates in D+/R− patients (9).

It is well established that, overall, D+/R− patients experience higher peak viral loads than D+/R+ or D−/R+ patients, indeed this is a key demonstration of the effectiveness of the CMV-specific immune response (14, 20, 21). Our analysis extends this demonstration by suggesting that the overall elevation in peak viral load in D+/R− patients is, in part, driven by the HLA-mismatched individuals. Nevertheless, in most cases, the scale of the effects that we observed was small. Hepatocytes express relatively low levels of HLA class I and class II molecules (22, 23), providing a plausible rationale for the minimal effect of HLA A and B mismatching. However, we cannot estimate the impact of expression of class I and II antigens on other cell types, such as Kupfer cells (for class II) and bile duct or vascular epithelia (for class I).

Given the data available for this study, it has not been possible to examine the impact of HLA micropolymorphisms or supertypes. We therefore recommend that other liver transplant centers analyze their data using similar methods, and consider pooling the results to increase sample size, which would allow finer mapping of HLA diversity than was available in our study.

Our data raise interesting questions about the pathogenesis of CMV in the liver transplant setting. Our measure of CMV replication is based on the detection of viral DNA in whole blood. However, we do not know the cellular origin of the virus that gives rise to this viremia. So, while the original source of CMV in the D+/R− patients is definitely the donor liver and the donor may reinfect a seropositive recipient in the D+R+ combination (4), liver cells may not be the major source of virus detected in blood.

It is conceivable that a small inoculum of liver-derived virus infects recipient cells in other organ systems leading to amplification and consequent seeding of viremia. Such producer cells would be susceptible to T cell-mediated immune control because viral antigens would be presented by recipient cells in the correct HLA context. This possibility is supported by work with murine CMV (MCMV) demonstrating that high-level MCMV replication in hepatocytes had a negligible impact on the viral load in blood, which was derived primarily from infected endothelial cells (24). If such a phenomenon were operating in the human system, the effects of HLA matching would be minimized. Such a scenario could resolve the apparent contradiction between our current observations and the correlation that we (and others) have documented previously in CMV-positive liver transplant patients between a polyfunctional T-cell response and control of viremia (7, 18, 19). It could also provide a potential explanation for the lack of association between cumulative viral load in whole blood and CMV hepatitis in our previous studies (25). Thus, it is possible that CMV replication in the liver produces abnormal aminotransferase levels as a consequence of viral lysis of the liver cells in HLA-mismatched individuals, whereas in the HLA-matched setting, a discrete CMV hepatitis may be driven predominantly by the immune destruction of liver cells as observed in hepatitis B infection. By analogy with the MCMV model, the progeny virus resulting from replication in donor cells would not be detected in whole blood, as the majority remains confined within the liver.

In conclusion, the results in this report are consistent with the view that, in a D+ setting, while the donor organ is the initial source of virus, cytotoxic T lymphocyte-mediated control of CMV replication in the liver has minimal impact on the overall immune control of CMV infection. These data have relevance to the use of adoptive transfer of immune committed T cells and/or immunization of recipients awaiting transplantation as potential ways of reducing donor-to-recipient transmission of CMV and CMV replication post transplantation.

## Supporting Information

**Table S1.** Number of individuals who developed high-level cytomegalovirus (CMV) viremia (>3000 genomes/mL) during follow up, according to donor (D)/recipient (R) CMV status and human leukocyte antigen (HLA) mismatch level. **Table S2.** Peak viral load (genomes/mL) according to donor (D) and recipient (R) cytomegalovirus status and human leukocyte antigen (HLA) mismatch level.
