# Supplementary material for: Effects of donor/recipient human leukocyte antigen mismatch on human cytomegalovirus replication following liver transplantation
Source: Transpl Infect Dis. 2015 Jan 9;17(1):25–32. doi: 10.1111/tid.12325 (PMC4345424; doi:10.1111/tid.12325)
Supplement: Supplementary file 1 [file tid0017-0025-sd1.doc]

**Number of individuals who developed high-level cytomegalovirus (CMV) viremia (> 3000 genomes/mL) during follow-up, according to donor (D)/ recipient (R) CMV status and human leukocyte antigen (HLA) mismatch level**

| HLA A |  |  |  |  |  |  |
| --- | --- | --- | --- | --- | --- | --- |
|  | HLA  mismatches | Total in  group | Viremia | | |  |
|  |  |  | Yes | % (95% CI) | | *P*-value* |
| D– R– | 0 | 6 | 0 | 0 | (0, 46) |  |
|  | 1 & 2 | 32 | 0 | 0 | (0, 11) | - |
| D– R+ | 0 | 10 | 1 | 10 | (0, 45) |  |
|  | 1 & 2 | 92 | 8 | 9 | (4, 16) | 1.00 |
| D+ R+ | 0 | 20 | 4 | 20 | (6, 44) |  |
|  | 1 & 2 | 81 | 19 | 23 | (15, 34) | 1.00 |
| D+ R– | 0 | 5 | 2 | 40 | (5, 85) |  |
|  | 1 & 2 | 28 | 20 | 71 | (51, 87) | 0.30 |
|  |  |  |  |  |  |  |
| HLA B |  |  |  |  |  |  |
|  | HLA  mismatches | Total in  group | Viremia | | |  |
|  |  |  | Yes | % (95% CI) | | *P*-value* |
| D– R– | 0 | 4 | 0 | 0 | (0, 60) |  |
|  | 1 & 2 | 34 | 0 | 0 | (0, 10) | - |
| D– R+ | 0 | 8 | 1 | 13 | (0, 53) |  |
|  | 1 & 2 | 94 | 8 | 9 | (0, 16) | 0.54 |
| D+ R+ | 0 | 12 | 1 | 8 | (0, 38) |  |
|  | 1 & 2 | 89 | 22 | 25 | (16, 35) | 0.29 |
| D+ R– | 0 | 4 | 2 | 50 | (7, 93) |  |
|  | 1 & 2 | 29 | 20 | 69 | (49, 85) | 0.59 |
|  |  |  |  |  |  |  |
| HLA DR |  |  |  |  |  |  |
|  | HLA  mismatches | Total in  group | Viremia | | |  |
|  |  |  | Yes | % (95% CI) | | *P*-value* |
| D– R– | 0 | 8 | 0 | 0 | (0, 37) |  |
|  | 1 & 2 | 30 | 0 | 0 | (0, 12) | - |
| D– R+ | 0 | 9 | 2 | 22 | (2, 60) |  |
|  | 1 & 2 | 93 | 7 | 8 | (3, 15) | 0.18 |
| D+ R+ | 0 | 20 | 4 | 20 | (6, 44) |  |
|  | 1 & 2 | 81 | 19 | 23 | (15, 34) | 1.00 |
| D+ R– | 0 | 6 | 1 | 17 | (0, 64) |  |
|  | 1 & 2 | 27 | 21 | 78 | (58, 91) | 0.01 |
| *Fisher's exact test.  CI, confidence interval.  *Table S1* | |  |  |  |  |  |
|  |  |  |  |  |  |  |
|  | |  |  |  |  |  |
|  |  |  |  |  |  |  |

**Peak viral load (genomes/mL) according to donor (D) and recipient (R) cytomegalovirus status and human leukocyte antigen (HLA)** mismatch level

| HLA A |  |  |  |  |
| --- | --- | --- | --- | --- |
|  |  |  |  |  |
|  | HLA  mismatches | Total in  group | Median peak viral load, copies/mL (IQR*) | *P*-value** |
| D– R+ | 0 | 3 | 1123 (551–74708) |  |
|  | 1 & 2 | 29 | 979 (477–3315) | 0.46 |
| D+ R+ | 0 | 9 | 2616 (1648–4140) |  |
|  | 1 & 2 | 42 | 2627 (713–5102) | 0.84 |
| D+ R– | 0 | 4 | 3540 (1575–7495) |  |
|  | 1 & 2 | 23 | 14706 (8131–68854) | 0.03 |
|  |  |  |  |  |
|  |  |  |  |  |
|  |  |  |  |  |
| HLA B |  |  |  |  |
|  |  |  |  |  |
|  | HLA  mismatches | Total in  group | Median peak viral load,(copies/mL (IQR*) | *P*-value** |
| D– R+ | 0 | 3 | 706 (499–74,708) |  |
|  | 1 & 2 | 29 | 1114 (477–3315) | 0.67 |
| D+ R+ | 0 | 4 | 1440 (256.5–14,619) |  |
|  | 1 & 2 | 47 | 2677 (779–5102) | 0.44 |
| D+ R– | 0 | 3 | 4838 (2241–20,979) |  |
|  | 1 & 2 | 24 | 12645 (7832–62,827) | 0.32 |
|  |  |  |  |  |
|  |  |  |  |  |
| HLA DR |  |  |  |  |
|  |  |  |  |  |
|  | HLA  mismatches | Total in  group | Median peak viral load, copies/mL (IQR*) | *P*-value** |
| D– R+ | 0 | 3 | 3371 (555–74,708) |  |
|  | 1 & 2 | 29 | 979 (477–2738) | 0.21 |
| D+ R+ | 0 | 10 | 1665 (344–3166) |  |
|  | 1 & 2 | 41 | 2677 (1114–5102) | 0.52 |
| D+ R– | 0 | 3 | 2241 (634–4838) |  |
|  | 1 & 2 | 24 | 13832 (8146–62,827) | 0.02 |
|  |  |  |  |  |
| *Interquartile range. | |  |  |  |
| **Kruskall–Wallis test. | |  |  |  |

Note: Data presented are for a subset of individuals with detectable viremia (i.e., viral load >200 genomes/mL).

*Table S2*
